# *Trichuris muris*: a model of gastrointestinal parasite infection

**DOI:** 10.1007/s00281-012-0348-2

**Published:** 2012-10-11

**Authors:** Joanna E. Klementowicz, Mark A. Travis, Richard K. Grencis

**Affiliations:** 1 Department of Surgery, The University of California San Francisco, San Francisco, CA USA; 2 Manchester Immunology Group and Wellcome Trust Centre for Cell-Matrix Research, The Faculty of Life Sciences, University of Manchester, Manchester, UK; 3 Immunology Group, The Faculty of Life Sciences, University of Manchester, Oxford Road, Manchester, M13 9PT UK

**Keywords:** *Trichuris muris*, Intestinal helminths, Resistance, Susceptibility, Innate lymphoid cells, Basophils

## Abstract

Infection with soil-transmitted gastrointestinal parasites, such as *Trichuris trichiura*, affects more than a billion people worldwide, causing significant morbidity and health problems especially in poverty-stricken developing countries. Despite extensive research, the role of the immune system in triggering parasite expulsion is incompletely understood which hinders the development of anti-parasite therapies. *Trichuris muris* infection in mice serves as a useful model of *T. trichiura* infection in humans and has proven to be an invaluable tool in increasing our understanding of the role of the immune system in promoting either susceptibility or resistance to infection. The old paradigm of a susceptibility-associated Th1 versus a resistance-associated Th2-type response has been supplemented in recent years with cell populations such as novel innate lymphoid cells, basophils, dendritic cells and regulatory T cells proposed to play an active role in responses to *T. muris* infection. Moreover, new immune-controlled mechanisms of expulsion, such as increased epithelial cell turnover and mucin secretion, have been described in recent years increasing the number of possible targets for anti-parasite therapies. In this review, we give a comprehensive overview of experimental work conducted on the *T. muris* infection model, focusing on important findings and the most recent reports on the role of the immune system in parasite expulsion.

## Introduction

Soil-transmitted helminth (STH) infections, mainly trichuriasis, ascariasis and hookworm, affect more than a billion people, especially in poverty-stricken areas in the developing world [1]. *Trichuris trichiura* alone is believed to infect almost 800 million people worldwide, with the majority being children [1]. Infected children show signs of malnutrition, stunted growth, intellectual retardation and educational deficits [2]. Moreover, infection during pregnancy increases the risk of maternal anaemia and reduces infant birth weight and survival [2]. The burden of diseases attributed to STHs is of great consequence to economic progress of developing countries, trapping affected people and whole communities in poverty. Therefore, increasing our understanding of how effector immune responses against STHs are induced and controlled is pivotal for the development of novel therapies.

In recent decades, studies on the gastrointestinal parasite *Trichuris muris*, a mouse model of *T. trichiura* infection in humans, have greatly contributed to our knowledge on components of immune responses responsible for resistance and susceptibility to infection. Research conducted on *T. muris* has presented us with novel explanations on how the immune system induces parasite expulsion, which could have broader application for new treatment development against soil-transmitted parasite infections.

## *T. muris*

### Life cycle

**Fig. 1:**
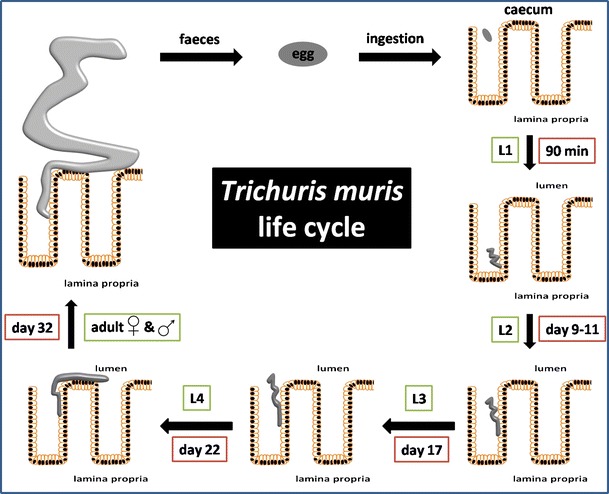
*Trichuris* *muris* life cycle. Infection occurs by the ingestion of infective eggs which hatch in the caecum 90 min post infection (p.i.) releasing the first larvae (*L1*). *L1* penetrate the caecum and proximal colon wall, dwell in the epithelial layer and undergo three more moults to *L2* (9–11 days p.i.), *L3* (17 days p.i.) and *L4* stage (22 days p.i.). By day 32 p.i. female and male adult forms of *T. muris* can be observed in the caecum and proximal colon of infected mice. Eggs, which leave the host organism with faeces, need 2 months to embryonate and become infective

Infection with *T. muris* occurs by the ingestion of infective eggs that accumulate in the caecum (Fig. 1). Ninety minutes post infection (p.i.), the first larvae (L1) hatch from eggs. Interestingly, egg interaction with the bacterial microflora of the gut is important for induction of parasite hatching [3]. Experimentally, using laboratory-derived strains of bacteria, this process has been shown to be dependent on bacterial type 1 fimbriae which normally facilitate mannose-sensitive adherence of bacteria to cells and mucosal surfaces. Culturing of *T. muris* eggs with *Escherichia coli* strain which lacks a gene cluster responsible for type 1 fimbriae expression resulted in severely impaired parasite hatching [3]. In addition, mice treated with antibiotics had reduced numbers of worms compared to untreated controls [3]. Whilst the precise species of bacteria responsible for hatching in vivo remains to be defined, the data do provide an explanation for why hatching occurs preferably in the caecum—the main site of the intestinal microflora.

Upon hatching, L1 penetrate the caecum and proximal colon wall, dwell in the epithelial layer and undergo three more moults to L2 (9–11 days p.i.), L3 (17 days p.i.) and L4 stage (22 days p.i.). Moults may occur at slightly different time points depending on the strain of the host. During larval development, the parasite moves from solely within the epithelial layer to extend into the gut lumen. By day 32 p.i., adult worms are observed in the caecum and proximal colon of infected mice. Interestingly, the anterior part of the worm is buried in parasite-modified epithelial cells which form a structure resembling ‘syncitial tunnels’. Tilney et al. [4] have shown via electron microscopy that *T. muris* lives in direct contact with modified epithelial cell cytoplasm. Boring of the parasite into epithelium causes the surrounding cells to rupture mainly by affecting the lateral wall of cells. The apical and basal surfaces of cells often remain intact leading to the creation of ‘tunnels’ in which the parasite dwells [4]. Eggs, which leave the host organism with faeces, need approximately 2 months to embroynate and become infective (reviewed in [5]).

### Host predispositions to infection—genetic background and gender

Early studies on outbred and inbred strains of mice suggested that the genetic background of the host was an important element contributing to observed variation in susceptibility to *T. muris* infection [5]. Indeed, it has been shown that genes within the H-2 allele of the major histocompatibility complex (MHC) and non-H-2 genes can affect resistance to *T. muris*. Studies on two groups of congenic strains have demonstrated that certain genetic backgrounds are more resistant than others (e.g. mice on the BALB genetic background are more resistant than mice on B10 background) even if they share the same H-2 haplotype [6]. Likewise, mice sharing certain ‘resistant’ H-2 alleles within the I-A region (H-2^q^, H-2^b^) expel parasites faster than mice having a ‘susceptible’ H-2 phenotype (H-2^k^, H-2^d^). The influence of these alleles was further modulated by the differences in the D end of the H-2 complex between these strains [7]. Therefore, the H-2 complex can affect expulsion kinetics; however, genes from outside the MHC complex play a dominant role in determining the outcome of *T. muris* infection in mice. This is especially evident in BALB/k and AKR mice, both sharing the H-2k haplotype, with the former generating a resistant-associated Th2 response followed by parasite expulsion and the latter succumbing to chronic infection accompanied by the development of a Th1 response [8]

Beside the genetic background influence on resistance to *T. muris* infection, the gender of the host also affects the efficiency of worm clearance. Studies by Bancroft et al. have shown that IL-4-deficient BALB/c male and female mice respond differently to *T. muris* infection, with males developing a chronic infection whereas females expel the worms (although later than wild-type controls) [9]. Further research has demonstrated that this difference in expulsion kinetics between genders is IL-13-dependent, since treatment of female IL-4 KO mice with anti-IL-13 antibody resulted in a lack of parasite clearance and administration of recombinant IL-13 restored a resistant phenotype in IL-4 KO male mice [9]. Interestingly, neutralization of IFN-γ resulted in normal parasite expulsion in both female and male IL-4 KO mice [10]. Similar differences in parasite expulsion have also been observed in TNF-α receptor KO mice (p55/p75 KO mice), where female mice are resistant to infection whereas males become chronically infected [11]. Moreover, similar to IL-4 KO mice, neutralization of IL-13 in female p55/p75 KO mice rendered them susceptible to infection and administration of recombinant IL-13 to male p55/p75 KO animals restored parasite expulsion [11]. Further studies on sex steroid hormones have revealed that male-associated dihydrotestosterone can decrease the ability of dendritic cells (DCs) to activate T cells and also skew T cell differentiation towards a Th1-type response via IL-18-dependent mechanisms [12]. Conversely, the female-related hormone 17-β-estradiol (E2) seems to enhance the generation of a Th2 response, at least in vitro [12]. These differences in sex hormones and their ability to affect the development of immune responses to *T. muris* should be considered a contributing factor in the variation in resistance to infection with this parasite. Taken together, both host genetic background and gender can greatly influence the type of immune responses generated against *T. muris* and subsequent parasite expulsion.

### Infective dose

Apart from host genetic background and gender, the size of infective dose and parasite genetics can also influence the variation in susceptibility to infection with *T. muris*. It has been demonstrated that decreasing the infective dose can alter the polarization of the immune response favouring the development of a susceptibility-associated Th1 immune response. Normally resistant BALB/k mice when infected with less than 40 *T. muris* eggs (low dose) instead of 400 eggs (high dose) develop chronic infection [13]. Moreover, only high dose infection can render mice resistant to subsequent high and low dose challenge infections [14].

In addition to differences in host immune responses to low and high antigen load, different isolates of *T. muris* can also elicit distinct reactions from the immune system of the host. There are three laboratory-used isolates of *T. muris*: E (Edinburgh), J (Japan) and S (Sobreda) isolates [15]. It has been shown that B10.BR, CBA and C57BL/10 mice, normally resistant to infection with both E and J isolates, develop chronic infection when infected with S isolate [15, 16]. This was related to increased Th1 and decreased Th2 responses in S isolate-infected mice reflected in higher levels of IFN-γ and Th1-associated IgG2a production [15, 16]. On the other hand, mice infected with E or J isolate produced Th2-associated IL-5 and had higher levels of Th2-associated IgG1 in the serum [16]. The same kinetics of expulsion has been also observed for C57BL/6 mice which expel E isolate normally but develop chronic infection when infected with S isolate [17]. Here susceptibility to *T. muris* has been associated with increased numbers of regulatory T cells (Tregs) in the gut of mice infected with S but not E isolate. It has been therefore suggested that Tregs can inhibit the development of protective immunity and promote chronic infection [17].

## Immune responses to *T. muris* infection

The type of immune response generated against *T. muris* is critical in mediating either susceptibility or resistance to infection. The development of a Th2-type of response is associated with fast parasite expulsion whereas a Th1 response is linked to establishment of chronic infection and increased immunopathology. The use of different mouse strains and gene knockout animals has been crucial in determining important cellular and molecular pathways of importance during *T. muris* infection (see Table 1 for summary). In this section, we discuss cells and molecules found to be important in the generation of immune responses to *T. muris* and their role in promoting and hampering parasite expulsion. .

**Table 1:** A summary of differences in immune responses to *T. muris* between different mouse strains

Strain	Description	Phenotype	Notes	References
AKR	Susceptible background	S	Develop Th1 response regardless of dose of infective eggs	[8]
Nude	Athymic mice, lack of adaptive immune responses	S	Adoptive transfer of CD4^+^ T cells results in parasite expulsion	[19]
SCID	Lack of V(D)J recombination, no T or B cells	S	Adoptive transfer of CD4^+^ T cells results in parasite expulsion	[23]
μMT KO	Lack of B cells	S	Th1 response development, resistance restored by neutralization of Th1-promoting IL-12	[48]
MHCII^CD11c^	MHCII expression restricted to CD11c^+^ cells	S	lack of Th2 development, resistance restored by neutralization of Th1-promoting IFN-γ	[33]
IL-4 KO (BALB/c)	Lack of IL-4, gender dependent	S ♂	In male mice resistance can be restored by IL-13 treatment.	[9], [10],
			Female mice expel slightly later than WT controls. Susceptibility can be induced by IL-13 neutralization.	
		R ♀	WT phenotype can be restored in both by INF-γ neutralization	
IL-25 KO	Lack of IL-25	S	lack of MMP^type2^ cells, impaired Th2 response development, resistance restored by adoptive transfer of MMP^type2^ cells	[29]
IL-10 KO	Lack of IL-10	S	death caused by sever immunopathology of the gut	[77]
Muc5ac KO	Lack of Muc5ac mucin	S	strong Th2 response, however, lack of Th2-regulated Mu5ac mucin production responsible for parasite expulsion	[88]
IL-4R KO	Lack of IL-4 and IL-13 receptor	S	no Th2 response	[90]
p55/p75 KO (C57BL/6)	Lack of TNF-α receptor, gender dependent	S ♂	In male mice resistance can be restored by IL-13 treatment.	[11]
		R ♀	In female mice susceptibility can be induced by IL-13 neutralization.	
TSLPR KO	Lack of TSLP receptor	S	Lack of Th2 response development, resistance restored by neutralization of Th1-promoting IFN-γ	[71]
C57BL/6	Dose dependent	S/R	Develop Th1 response when infected with low dose of eggs	[14]
BALB/k			Develop Th2 when infected with high dose of eggs.	[13]
BALB/c				[89]
WSX-1 KO	Lack of IL-27 receptor	R	Lack of Th1 development, IL-27 signalling responsible for triggering Th1 response	[60]
CCL11/IL-5 double KO	Lack of CCL11 and IL-5, no eosinophils	R	Lack of eosinophils has no effect on parasite expulsion	[55]
RELMβ KO	Lack of RELMβ	R	Decreased production of T cell-derived IFN-γ and TNF-α	[91]

*S* susceptible, *R* resistant, *WT* wild type, ♀ female mice, ♂ male mice

### Components of the immune response to *T. muris*

#### T cells

T cells were shown to be important in mediating *T. muris* expulsion as early as 1983 by Lee et al. In these experiments, transfer of T cell-enriched but not B cell-enriched populations from *T. muris* infected donors into naive recipients transferred immunity to infection [18]. In addition, it has been demonstrated that congenitally athymic mice (Nude mice), which lack T cells, are susceptible to *T. muris* infection [19]. However, worm expulsion could be observed in Nude mice after splenocyte transfer. Moreover, transfer of mesenteric lymph node (MLN) cells or thymocytes also partially restored a resistant phenotype in these mice [19]. Studies on different subpopulations of T cells have brought further insight into the importance of T cells during *T. muris* infection, showing that depletion of CD4^+^ T cells but not CD8^+^ T cells or NK1.1^+^ natural killer T cells with neutralizing antibodies resulted in susceptibility to infection [20–22]. Additionally, adoptive transfer of CD4^+^ T cells from resistant BALB/c mice into severe combined immunodeficiency (SCID) mice, which lack the adaptive arm of the immune system and therefore are susceptible to infection, resulted in worm expulsion proving that CD4^+^ T cells, and not CD8^+^ T cells or B cells, are crucial for the development of protective immunity to *T. muris* [23]. It has been further demonstrated that CD4^+^ T cells are most effective against larval stages of the parasite and that they act locally at the site of infection, since inhibition of the gut homing receptors β7 and αE integrins and the gut homing ligand MAdCAM-1 completely abrogates the ability of transferred CD4^+^ T cells to expel infection in SCID mice [24, 25]. Interestingly, inhibition of CCR6 and CXCR3 chemokine receptors, which are proposed to be important in gut homing by T cells and are the most abundant chemokine receptors expressed by CD4^+^ T cells in the MLN, did not prevent expulsion of infection [25].

Analysis of intestinal intraepithelial lymphocytes (IELs) in the large intestine of *T. muris-*infected resistant BALB/c and susceptible AKR mice have shown that at the time of expulsion (around day 21 post infection) BALB/c mice have increased numbers of CD4^+^ IELs whereas IELs in AKR animals are predominantly CD8^+^ [26]. Interestingly, normally resistant mice become more susceptible to infection with age due to a decreased ability of CD4^+^ T cells to respond to stimulation and to polarize into Th2 cells [27]. Taken together, the development of protective immunity against *T. muris* depends almost completely on CD4^+^ T lymphocytes.

#### Innate lymphoid cells

The hypothesis that early immune events may be important in mounting protective responses during *T. muris* infection is in agreement with recent reports on novel innate cell populations, driven mainly by IL-25 and IL-33 production, which can serve as a source of Th2 cytokines early during GI parasite infections [28]. A new, IL-25-induced cell type called multi-potent progenitor type 2 (MMP^type2^) has been suggested to be important in resistance to *T. muris* infection [29]. MMP^type2^ cells are linage^−^ Sca-1^+^ c-kit^int^ and can be found in all compartments of gut-associated lymphoid tissue (GALT) such as MLNs, Payer’s patches and caecal patches but not in the spleen or bone marrow of IL-25-treated mice. As their name suggests, they can give rise to many different cell populations, i.e. monocyte, macrophages, mast cells and basophils both in vitro and in vivo. They have also been shown to promote T cell proliferation and differentiation toward a Th2 phenotype in vivo. In terms of *T. muris* infection, mice deficient in IL-25 (IL-25 KO mice) are susceptible to infection, showing reduced Th2 cytokine production and impaired mucin responses, with the phenotype being reversed by MPP^type2^ cell transfer [29]. Thus, IL-25 KO mice treated with IL-25-elicited MMP^type2^ cells had reduced worm numbers at day 20 post infection compared to the untreated controls. Consistently, treated mice showed increased production of resistance-associated IL-4, IL-5 and IL-13 in the MLNs and higher IgG1 levels in the serum [29]. Therefore, since MMP^type2^ cells have been shown to promote development of a Th2-type response, they can be responsible for triggering a resistance-associated T cell response essential for *T. muris* expulsion. Furthermore, recently described nuocytes [30], natural helper cells [31] and innate type 2 helper cells [32], have been shown to play similar essential roles in early induction of Th2 immunity during *Nippostrongulus brasiliensis* infection. Hence, it remains to be seen whether these novel innate cell types play a similar role to MPP^type2^ cells during *T. muris* infection.

#### Basophils versus dendritic cells in priming Th2 responses during infection

Basophils can be found in draining lymph nodes upon activation of the immune system and have recently been shown to express MHC class II and produce Th2-inducing cytokines such as TSLP and IL-4 [33–35]. IL-4-producing basophils as a source of Th2-inducing cytokines could facilitate DC-mediated Th2 differentiation [36]. In addition to acting as accessory cells aiding DC-mediated development of Th2 responses, it has been implied in recent years that basophils can also serve as professional antigen presenting cells to directly induce Th2-mediated immunity [33, 35].

Indeed, there is now data indicating that basophils can play an important role in induction of a Th2 response during parasitic infection, including *T. muris* infection. Depletion of FcεRI^+^ cells (which include basophils) results in reduced Th2 responses in the intestine and a trend for increased worm burden during *T. muris* infection [33]. Furthermore, reduced levels of IL-5 and IL-13 and lack of expulsion in TSLP receptor-deficient mice (TSLPR KO mice) is linked with decreased numbers of basophils in these animals during acute *T. muris* infection [37]. Interestingly, adoptive transfer of basophils into TSLPR KO mice leads to a reduction in worm numbers, although expulsion is still delayed compared to control animals [37].

Interestingly, restriction of MHCII expression to DCs is not sufficient to generate Th2 cytokine production and results in lack of parasite expulsion [33], suggesting that DCs are not required for generation of Th2 responses during *T. muris* infection. Additional treatment of mice with MHC class II restricted to DCs with IFN-γ neutralizing antibody led to the restoration of a resistant phenotype [33], suggesting that DCs are capable of triggering Th2 response during *T. muris* infection if Th1 development is abrogated. However, in subsequent work using different parasite infections and alternative strategies to abrogate the function of basophils and DCs, DCs were found to be crucial in the generation of parasite-induced Th2 responses. Thus, several studies have shown that basophil depletion has no effect on the development of Th2 responses and parasite clearance during primary infection with *N. brasiliensis* [38], although basophils do appear to be involved in responses to secondary infection [39]. Similarly, depletion of basophils during *Schistosoma mansoni* infection does not alter the development of Th2 responses [40, 41]. Instead, depletion of DCs during *S. mansoni* infection impairs the Th2 response [40]. Thus, whether the observed lack of requirement for DCs in the generation of Th2 responses during *T. muris* is due to the particular models used by Perrigoue et al. [33] or is a specific observation for this parasite requires further investigation. Moreover, it is still not clear whether basophils can serve as an antigen presenting cells during parasite infection.

Although there is some controversy about the importance of DCs in triggering Th2 responses during *T. muris* infection, evidence exists that susceptibility/resistance to infection is linked to differences in DC phenotype. Thus, mice that are resistant to infection have faster DC mobilization to the site of parasite infection than susceptible animals [42]. Th2-inducing DCs often do not demonstrate an up-regulation of surface activation markers and cytokine production upon antigen encounter [43]. However, in contrast, during *T. muris* infection DCs from resistant mice show higher expression of CD80/86, MHCII and CCR7 and lower endocytic activity than DCs from susceptible mice, indicating that DCs from resistant animals mature faster. Moreover, epithelial cells from resistant animals have higher expression of chemokines such as CCL2, CCL3, CCL5, CCL20 and TSLP early during infection which correlates with the rapid mobilization of DCs to the large intestine [42]. Indeed, mice treated with antibodies against CCL5/CCL20 chemokines showed decreased DC mobilization to the large intestine [42].

Furthermore, increased frequency of CD103^+^ DCs, which have been shown to have tolerogenic properties due to elevated production of retinoic acid [44, 45] and expression of the TGF-β-activating integrin αvβ8 [46], have been reported in the lamina propria of resistant mice [47]. However, since CD103 KO mice have similar expulsion kinetics during both acute and chronic infection with *T. muris*, expression of CD103 by these cells seems to be dispensable for the development of protective immunity against this parasite [47].

#### B cells and antibodies

B cells and B cell-produced antibodies have been suggested in the past to be involved in the generation of protective immunity against *T. muris* infection [20, 48]. IgG- and IgA-producing cells have been detected in MLNs as early as days 14 and 21 post infection [49, 50]. Furthermore, resistant and susceptible animals differ in the type of produced antibody class showing elevated levels of IgG1 and IgG2a, respectively [49]. These differences in antibody responses can be correlated with the development of distinct T cell-mediated responses linked with different cytokine profiles, namely the Th2 response in resistance and Th1 in susceptibility to *T. muris* infection [50].

In addition, it has been demonstrated that B-cell deficient mice (μMT mice) are susceptible to *T. muris* infection and that their resistant phenotype can be restored by adoptive transfer of B cells or administration of IgG from resistant mice infected with the parasite [48]. However, adoptive transfer of CD4^+^ T cells alone into SCID mice results in successful expulsion of *T. muris*, indicating that expulsion can occur in the absence of B cells [23]. Indeed, treatment of μMT mice with anti-IL-12 antibody, which blocks the development of the susceptibility-associated Th1 response, also leads to parasite expulsion in these animals. Therefore, it seems that facilitating the development of Th2 responses via blocking of IL-12 can result in antibody-independent expulsion of *T. muris* [48]. Thus, the role of B cells and antibody during *T. muris* infection requires further investigation.

#### Mast cells

Mast cell infiltration and activation are one of the hallmarks of parasitic infections. Indeed, mice infected with *T. muris* show an increase in mast cell numbers upon infection [51]. However, mast cell appearance at the site of infection in many cases does not correlate with parasite expulsion; for example, in NIH mice worms are expelled 10 days before mastocytosis develops [51]. On the other hand, studies have shown that transfer of IL-9-secreting T cells into mice infected with *T. muris* results in increased mast cell numbers at the site of infection, elevated serum levels of mMCP-1 (mouse mast cell protease 1) and faster parasite expulsion [52]. Moreover, naturally mast cell-deficient mice (W/Wv mice) demonstrate delayed *T. muris* expulsion [53]. However, since their wild-type counterparts can expel parasite without any sign of mastocytosis, it seems that the role of mast cells in generating protective immunity against *T. muris* is minor [53]. Furthermore, depletion of mast cells using neutralizing antibody against c-kit receptor, a molecule critical for mast cell development, had no effect on parasite expulsion [54]. Therefore these cells are believed to be dispensable for the generation of protective immunity against *T. muris*.

#### Eosinophils

Increased numbers of eosinophils are characteristic of parasitic helminth infections. Mice resistant to *T. muris* infection have elevated numbers of these cells in the colon [55] and MLNs [56] during infection. The induction of eosinophilia during *T. muris* infection is under the control of IL-5 and the chemokine CCL11 which work in synergy to recruit eosinophils [55]. CCL11 KO mice have reduced numbers of these cells in the colon and double knockout mice of CXCL11 and IL-5 completely lack eosinophilia [55]. Moreover, neutralization of eosinophils with anti-IL-5 antibody also reduced recruitment of these cells to the site of infection [54]. It has been recently show that eosinophils found in the MLNs of resistant mice have an activated phenotype and can produce IL-4 [56]. It is possible, therefore that they could contribute to the development of the resistance-associated Th2 response. However, reduction or depletion of eosinophils during *T. muris* infection has no effect on the development of the Th2 response and parasite expulsion [54–56] indicating that eosinophils are dispensable for generation of the protective immune response against *T. muris*.

#### Cytokines

##### Susceptibility

Both humans and wild-type animals are usually susceptible to infection with gastrointestinal parasites suggesting that worms are capable of modulating immune responses of their host to prevent expulsion. Indeed, it has been shown that development of an inappropriate Th1 response leads to chronic infection. Susceptible mouse strains produce high levels of IFN-γ, IL-12 and IL-18, cytokine characteristic of a Th1 response [5] and IFN-γ depletion in normally susceptible animals renders them resistant [57]. Similarly, resistant mice treated with IL-12 develop chronic infection [58]. Resistance is also associated with decreased production of the Th1-inducing cytokine IL-18 [59]. However, since treatment of infected mice with recombinant IL-18 resulted in impaired IL-4 and IL-13 production but did not affect IFN-γ levels, it seems that IL-18 could have a direct negative effect on the Th2 response during *T. muris* infection [59]. In addition, neutralization of IL-18 in susceptible male IL-4 KO mice restored the ability of these mice to expel the parasite which was associated with increased production of Th2-related cytokines and a decrease in the Th1 response [10]. Interestingly, anti-IL-18 treatment had no effect on female IL-4 KO animals which normally show mildly delayed *T. muris* clearance [10].

Additionally, mice which lack the IL-27 receptor WSX-1 (WSX-1 KO mice) do not develop chronic infection. IL-27 and WSX-1 are believed to interact in the early stages of infection to trigger Th1 responses in susceptible animals. WSX-1 KO animals have increased production of Th2-associated cytokines (IL-4, IL-9, IL-13) and decreased levels of Th1-associated cytokines (IFN-γ, IL-12p40) [60]. Interestingly, it has also been reported that *T. muris* may produce an IFN-γ-like molecule which could potentially contribute to regulation of the host immune response and promotion of parasite survival [61]. Of note, prolonged inflammatory responses observed during chronic *T. muris* infection lead to the development of host-detrimental immunopathology (e.g. colitis) and carry a risk of exacerbating bystander immune responses to different agents. For example, it has been shown that chronic *T. muris* infection resulted in systemic up-regulation of pro-inflammatory mediators, i.e. IFN-γ, TNF-α and IL-17, leading to augmented brain injury in a mouse model of ischemia-induced stroke [62]. Chronically infected animals showed accelerated platelet aggregation in the brain capillaries, increased matrix metalloproteinase activation and microvascular injury. This exacerbated brain damage could be reversed by CCL5 (RANTES) neutralization suggesting a role for this Th1-related chemokine in amplifying pro-inflammatory responses systemically [62].

##### Resistance

Resistance-associated cytokines are usually produced against high-dose *T. muris* infection and lead to parasite expulsion by triggering expulsion mechanisms such as increased epithelial cell turnover, mucin production and muscle hypercontractility. Cytokines associated with protective immunity to *T. muris* infection are Th2-type cytokines. Two cytokines that play a major role in the resistance to infection are IL-4 and IL-13 [63]. Mice lacking either IL-4 or IL-13 are susceptible to infection with a high dose of *T. muris* which is expelled by wild-type animals [64]. Moreover, expulsion of *T. muris* infection can be stimulated in IL-4-deficient male mice by IL-13 administration [9]. In addition, female IL-4 KO mice on a BALB/c background which, unlike males, can clear a high dose infection of *T. muris* (although delayed compared to wild-type mice) become susceptible after IL-13 neutralization, indicating a dominant role for IL-13 in immunity against *T. muris* [9]. Both CD4^+^ T cells and DX5^+^ NK cells have been shown to be a source of IL-13 in IL-4 KO mice, although only depletion of the former results in complete abrogation of parasite clearance [10].

As mentioned before, male but not female TNF-α receptor deficient mice are susceptible to *T. muris* infection, a phenotype that can be restored by IL-13 administration [11]. Moreover, susceptible IL-4 KO mice treated with TNF-α are able to clear infection [65]. However, it has been observed that TNF-α can also promote the development of stronger Th1 response in normally susceptible mice [66]. Therefore, TNF-α is believed to enhance an ongoing immune response, either Th1 or Th2, during infection but is not essential for the development of immunity.

It also has been reported that IL-9 is significant in early development of a protective immune response against *T. muris*, since adoptive transfer of IL-9-producting T cells into susceptible mice results in faster parasite expulsion, increased intestinal mast cell infiltration and mMCP-1 levels [52]. Indeed, the peak of IL-9 production correlates with worm expulsion in an acute model of *T. muris* infection [52]. Furthermore, immunization of mice with IL-9-OVA complex, which leads to production of IL-9-neutralizing antibodies in treated animals, results in impaired parasite expulsion and decreased blood eosinophilia [67] further indicating an important role for this cytokine in promoting resistance to *T. muris*.

Other cytokines important in the development of the protective immune response in early stages of *T. muris* infection are IL-25 and IL-33 [68, 69]. As described previously, both of these cytokines can induce innate lymphoid cells which play an important role in the generation of resistance-associated immunity against parasites [69]. More specifically, IL-25 can induce generation of GALT-specific MMP^type2^ cells which have been shown to promote Th2 responses and *T. muris* expulsion [29]. In addition, IL-33 can induce production of IL-4, IL-9 and IL-13, cytokines associated with a Th2-type immune response, but can also cause increased gut pathology [68]. Mice resistant to *T. muris* infection produce more IL-33 at day 3 post infection compared to susceptible animals. Furthermore, susceptible mice treated with recombinant IL-33 early during *T. muris* infection become resistant [68]. However, the same treatment applied later during infection (chronic infection) does not result in worm expulsion. IL-33-mediated resistance seems to be T cell-dependent because IL-33-treated SCID mice remain susceptible to infection despite developing gut pathology [68].

In addition to these findings, increased production of IL-33 early during infection in resistant strains of mice has been correlated with increased production of the Th2-inducing cytokine TSLP [68]. Indeed, disruption of the TSLP-TSLPR interaction results in impaired Th2 cytokine production and susceptibility to *T. muris* [70, 71]. This ineffective parasite expulsion has been associated with increased production of pro-inflammatory cytokines such as IL-12/23 (detected by enhanced expression of the IL-12 and IL-23 common subunit p40), IFN-γ and IL-17A, and more severe gut inflammation [71]. Neutralization of IFN-γ resulted in restoration of a resistant phenotype in TSLPR KO mice [71] indicating an important role for TSLP in early priming of the immune response against *T. muris* towards the resistance-associated Th2 response.

In summary, IL-4 and/or IL-13 accompanied by a range of Th2-related cytokines induce changes in intestinal environment such as hypercontractility of gut muscle, increased mucus production and turnover of epithelial cells which contribute to efficient expulsion of *T. muris* (discussed further in the “Mechanisms of expulsion” section).

### Regulation of the immune response during infection

Prolonged infection with gastrointestinal parasites, if not properly regulated, can result in severe damage to surrounding tissues. Immunopathology associated with susceptibility to *T. muris* infection can be characterised by severe transmural inflammation of the colon. Mucosal and submucosal inflammation results in destruction of normal crypt architecture and a subsequent wasting disorder [72]. Interestingly, changes in the gut physiology and architecture during chronic *T. muris* infection are similar to those observed during inflammatory bowel disease (IBD). In addition, phenotypic and translational similarities between chronic *T. muris* infection and mouse models of IBD and human IBD have been recently shown by transcriptional profiling studies [72]. It is somehow surprising therefore that in recent years clinical studies have shown that repeated oral administration of *Trichuris suis* eggs, a related parasite which normally causes trichuriasis in pigs, leads to remission of IBD symptoms in some patients [73, 74]. However, the safety of such practices has been questioned, with worries about the potential development of persistent chronic infection with *T. suis* [75]. Nonetheless, the immune response generated against parasites is believed to have a regulatory effect on responses to unrelated antigens such as bacterial flora which is a likely causative agent of IBD [76]. Therefore, investigating the mechanisms behind such regulation may eliminate potentially harmful elements of infection, leading to the development of new therapies for bowel disorders.

However, it is still unclear how the immune system is regulated during *T. muris* infection. A cytokine that plays an important role in resistance to *T. muris* infection is the regulatory molecule IL-10, with mice deficient in IL-10 becoming susceptible to *T. muris* infection and developing severe and fatal intestinal pathology [77]. In addition, TGF-β seems to be significant in regulating responses to *T. muris*. Recent studies have shown that CD4-dnTGF-βRII mice, whose CD4^+^ T cell have reduced ability to respond to TGF-β due to expression of truncated version of TGF-β receptor II (dnTGF-βII), have increased worm burden and down-regulated levels of IL-4 and IL-9 but normal IL-13 production [78]. It has also been shown that Tregs may play an important role in *T. muris* infection. A specific isolate of *T. muris*, isolate S, causes chronic infection in normally resistant C57BL/6 mouse strain that is associated with increased numbers of Foxp3^+^ Tregs in the gut of these mice [17]. Moreover, treatment with antibodies against GITR (glucocorticoid-induced TNF-α–related) molecule, which target Treg function, results in decreased worm burden and modulation of the immune response [17]. Interestingly, stimulation of bone marrow-derived macrophages and dendritic cells with S isolate excretory-secretory antigen results in increased production of IL-6 and IL-10 [79]. Since IL-6 has been shown to up-regulate IL-10 production [80] and IL-10 is important in controlling the development of fatal pathology during *T. muris* infection [77], it is possible that the S isolate of *T. muris* stimulates a more regulatory environment to aid its survival. Moreover, a relatively new subset of regulatory cells called iT_R_35 (CD4^+^Foxp3^−^Ebi3^+^p35^+^IL-10^−^TGF-β^−^) has been proposed to be important in regulating immune responses during *T. muris* infection [81]. These cells are highly suppressive in vitro and in vivo in an IL-35-dependent but IL-10- and TGF-β-independent manner, and can be detected in the large intestine during *T. muris* infection [81]. Taken together, regulatory cells and cytokines seem to be important in modulating effector immune responses during *T. muris* infection. However, more work is required to further assess regulatory mechanisms operating during *T. muris* infection.

## Mechanisms of expulsion

### Epithelial cell turnover

**Fig. 2:**
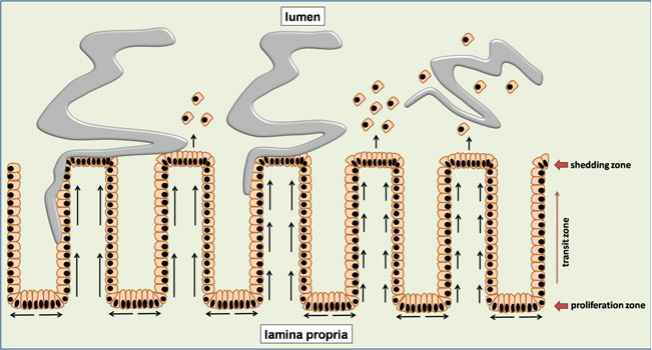
Epithelial cell turnover. Epithelial cells proliferate at the bottom of the crypt in the proliferation zone and subsequently migrate up the crypt through transit zone. When they reach the top of the crypt (*shedding zone*), they are removed. In resistance, mice infected with *T. muris* have accelerated epithelial cell turnover hindering worm ability to stay in the crypts attached to the epithelium. With faster epithelial cell turnover the parasite is moved to the top of the crypt, detached from the epithelium with shedded epithelial cells and subsequently expelled

Chronic infection with *T. muris* has been associated with crypt hyperplasia accompanied by both increased epithelial cell proliferation and apoptosis [82]. Both of these processes seem to be controlled by the pro-inflammatory cytokine IFN-γ and are believed to counterbalance each other in an attempt to control excessive crypt elongation in chronically infected animals [82, 83]. On the other hand, such dramatic changes are not observed in the gut of resistant mice. It has been demonstrated that resistant animals have accelerated epithelial cell turnover, a mechanism which is directly linked to faster parasite expulsion [84]. These findings led to a model referred to as the ‘epithelial escalator’, where epithelial cells move from the bottom of the crypt (proliferation zone) to its top (shedding zone), moving the parasite embedded in the epithelial layer towards the lumen where the epithelium and parasite are shed (Fig. 2). Therefore, mice that up-regulate epithelial cell turnover earlier during the infection (e.g. BALB/c) have an advantage over animals which fail to do so (e.g. AKR mice) [84]. Interestingly, differences in epithelial cell turnover between resistant and susceptible mice are due to differences in their immune responses and cytokine profiles, with Th1 and Th2 responses associated with down- and up-regulation of epithelial turnover speed, respectively. Studies in IL-4 KO and IL-13 KO mice have shown that acceleration in epithelial cell turnover is IL-13-dependent but IL-4-independent. On the other hand, IFN-γ and CXCL10 (IFN-γ-induced protein 10), both associated with a Th1 response and susceptibility to *T. muris* infection, seem to be responsible for epithelial cell turnover down-regulation [84]. Both AKR and SCID mice when treated with anti-CXCL10 antibodies showed an earlier up-regulation in epithelial cell turnover and parasite expulsion, further highlighting the importance of this mechanism in *T. muris* clearance [84]. In addition, recent studies have suggested a role for indoleamine 2,3-dioxygenase (IDO), an enzyme responsible for tryptophan degradation that is up-regulated during chronic *T. muris* infection, in controlling epithelial cell turnover ratio [85]. Normally susceptible SCID mice when treated with IDO inhibitor demonstrated faster epithelial cell turnover and parasite expulsion suggesting that IDO can also directly affect turnover upon *T. muris* infection [85]. Taken together, these data underline epithelial cell turnover as a major mechanism of *T. muris* expulsion.

### Mucin production by goblet cells

Goblet cells are major producers of mucins (the major protein component of mucus) and form an important element of the innate defence in the gastrointestinal tract. Goblet cell hyperplasia is observed during *T. muris* infection in both resistance and susceptible animals [86]. However, recent work by Hasnain et al. has shown that there is a significant variation in the type of mucin produced by intestinal goblet cells between resistant and susceptible mice [87–89]. Up-regulation of the mucin Muc2 was only observed in resistant animals and correlated with parasite expulsion. Moreover, Muc2-deficient mice demonstrated delayed parasite clearance compared to their wild-type counterparts [87]. Interestingly, Muc5ac, a mucin normally produced in airways and stomach but not the intestine, was also detected in resistant animals shortly before *T. muris* expulsion [87, 88]. Mice deficient in Muc5ac were susceptible to infection despite generating a strong Th2 response and stayed unable to expel the parasite even after treatment with IFN-γ neutralizing antibody which further enhanced the Th2 response [88]. Interestingly, treatment of adult *T. muris* worms with Muc5ac had detrimental effect on worm viability, as measured by parasite ATP levels [88]. These results indicate that certain mucins can have a direct damaging effect on worms.

The physical properties of the mucus barrier also changes during infection and correlates with responses to infection. Thus, the mucus barrier in resistant animals is less permeable, thicker and more highly charged than in susceptible mice [87, 89]. The intermediate barrier, rich in glycoproteins, shows higher levels of Muc4, Muc13 and Muc17 upon acute infection with *T. muris*. Interestingly, higher expression of Muc17 has also been observed in chronically infected animals [89]. Alteration in mucin glycosylation has also been reported between resistant and susceptible animals with the former showing higher expression of D-GalNAc glycan at the time of parasite expulsion [89]. Additionally, resistant animals show activation of the transcription factors atonal homolog 2 (Math-1) and SAM pointed domain containing ETS transcription factor (Spdef) which promote stem cell differentiation towards a secretory cell phenotype [89]. Hence, changes in the type, amount and state (charge and glycosylation) of mucin appear important in mediating immunity to *T. muris*.

Another goblet cell-derived molecule, resistin-like molecule β (RELMβ), has also been associated with immunity to *T. muris* infection with increased levels of this protein found in resistant animals [90]. Induction of RELMβ correlates with the production of Th2 cytokines. Indeed, IL-4 receptor KO mice, which lack both IL-4 and IL-13 signal transduction, but not IL-4 KO mice demonstrate impaired RELMβ expression, indicating an important role for IL-13 but not IL-4 in inducing RELMβ production [90]. It has been also suggested that RELMβ can have a direct negative effect on worms by affecting parasite chemosensory apparatus and thus impairing chemotaxis [90]. However, since RELMβ KO mice expel acute *T. muris* infection normally, RELMβ seems to be dispensable for the generation of Th2 response in resistant animals [91].

Conversely, studies have shown that RELMβ can actually promote chronic *T. muris* infection development [91]. Thus, RELMβ can facilitate development of a Th1 response via activation of intestinal macrophages which produce pro-inflammatory cytokines such as IL-12/23, IL-6 and TNF-α [91]. RELMβ KO mice show reduced intestinal inflammation associated with decreased production of T cell-derived IFN-γ and TNF-α, and fail to establish chronic infection [91].

Taken together, goblet cells and their products are important elements of the host immune responses against *T. muris* and seem to be indispensable for effective parasite clearance from the intestine.

### Muscle hypercontractility

It has been demonstrated that increased contractility of smooth muscle cells lining the wall of the intestine can be an important mechanism of gastrointestinal parasite expulsion. Studies on the small intestine-dwelling parasite *Trichinella spiralis* have shown that parasite expulsion is associated with muscle hypercontractility, via a STAT-6 and CD40-CD40L signalling-dependent mechanism controlled by the immune system [92, 93]. Interestingly, increased muscle contractility has been also related to increased resistance to *T. muris* infection [94]. This process has been reported to be controlled by IL-9, an important cytokine in resistance to *T. muris*. Indeed, neutralization of IL-9 by antibody treatment or by immunization with the OVA-IL-9 complex resulted in decreased smooth muscle contractility and the lack of parasite expulsion [94]. Moreover, susceptible AKR mice chronically infected with *T. muris* have been reported to have decreased muscle contractility which could be partially restored by treatment with the immunosuppressive drug dexamethason [95]. Thus, smooth muscle hypercontractility appears to be an important immune-mediated mechanism of *T. muris* expulsion.

**Fig. 3:**
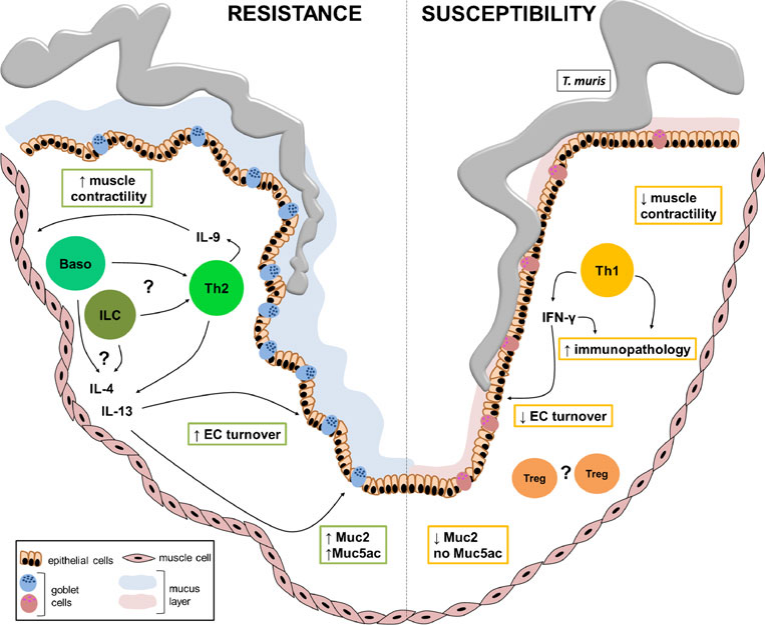
Mechanisms of *T. muris* expulsion. In resistance, generation of a Th2-type of a response is characterised by increased production of IL-4, IL-9 and IL-13. Basophils (*Baso*) and innate lymphoid cells (*ILC*) have been suggested to act as an early source of Th2 cytokines and to facilitate a Th2-type response development. Both increased epithelial cell (*EC*) turnover and increased production of mucins have been shown to be IL-13-dependent. Up-regulation of mucin secretion results in the thickening of a mucus layer which makes it more difficult for the parasite to stay attached to the epithelium. Also, mucus of resistant animals is rich in mucins such as Muc5ac which have a direct detrimental effect on worm viability. Moreover, IL-9 induces an increase in muscle contractility in the gut facilitating parasite expulsion. On the contrary, in susceptibility development of a Th1 response and production of IFN-γ result in slower EC turnover and muscle contractility, decreased Muc2 and lack of Muc5ac production. Furthermore, an exacerbated Th1 response eventually leads to immunopathology development resembling colitis. In addition, regulatory T cells (Treg) have been implicated in promoting susceptibility to infection with *T. muris*

Taken together, faster epithelial cell turnover, increased mucus production and muscle contractility are three known and characterised as immune-mediated mechanisms of *T. muris* expulsion (Fig. 3).

## Conclusion

*T. muris*, as a mouse model of *T. trichiura* in humans, has contributed greatly to an increase in our understanding of the relation between gastrointestinal parasite and its host in terms of generated immune responses and expulsion mechanisms. Moreover, knowledge acquired while studying *T. muris* model may be applied to other soil-transmitted gastrointestinal parasite infections. This is especially important since despite their evident public-health importance, soil-transmitted gastrointestinal infections remain largely neglected by medical and international societies. Part of the problem is that most affected individuals are also the world’s most impoverished and since helminth infections cause chronic illnesses most people cannot afford the prolonged and repeated treatment. Moreover, people in endemic areas are usually infected with more than one parasite causing more obscure and hard to diagnose clinical presentation [2]. Treatment of gastrointestinal infections is limited to chemotherapy with antihelminthics, with widespread and frequent use carrying the risk of the parasites developing drug resistance [96]. Moreover, even though progress in vaccine construction has been made in recent years [97], insufficient knowledge of how immune responses are induced and regulated during parasite infections impairs fast advancement in this field. Therefore, it is essential to increase our understanding of immune mechanisms responsible for controlling the development and expulsion of helminth infections which can subsequently lead to development of novel therapies.

## References

[1] WHO (2006) Preventive chemotherapy in human helminthiasis: coordinated use of anthelminthic drugs in control interventions: a manual for health professionals and programme managers. Available:http://whqlibdoc.who.int/publications/2006/9241547103_eng.pdf.

[2] Bethony J, Brooker S, Albonico M, Geiger SM, Loukas A (2006). Soil-transmitted helminth infections: ascariasis, trichuriasis, and hookworm. Lancet.

[3] Hayes KS, Bancroft AJ, Goldrick M, Portsmouth C, Roberts IS (2010). Exploitation of the intestinal microflora by the parasitic nematode Trichuris muris. Science.

[4] Tilney LG, Connelly PS, Guild GM, Vranich KA, Artis D (2005). Adaptation of a nematode parasite to living within the mammalian epithelium. J Exp Zoolog Part A Comp Exp Biol.

[5] Cliffe LJ, Grencis RK (2004). The Trichuris muris system: a paradigm of resistance and susceptibility to intestinal nematode infection. Adv Parasitol.

[6] Else K, Wakelin D (1988). The effects of H-2 and non-H-2 genes on the expulsion of the nematode Trichuris muris from inbred and congenic mice. Parasitology.

[7] Else KJ, Wakelin D, Wassom DL, Hauda KM (1990). The influence of genes mapping within the major histocompatibility complex on resistance to Trichuris muris infections in mice. Parasitology.

[8] Else KJ, Hultner L, Grencis RK (1992). Modulation of cytokine production and response phenotypes in murine trichuriasis. Parasite Immunol.

[9] Bancroft AJ, Artis D, Donaldson DD, Sypek JP, Grencis RK (2000). Gastrointestinal nematode expulsion in IL-4 knockout mice is IL-13 dependent. Eur J Immunol.

[10] Hepworth MR, Grencis RK (2009). Disruption of Th2 immunity results in a gender-specific expansion of IL-13 producing accessory NK cells during helminth infection. J Immunol.

[11] Hayes KS, Bancroft AJ, Grencis RK (2007). The role of TNF-α in Trichuris muris infection I: influence of TNF-α receptor usage, gender and IL-13. Parasite Immunology.

[12] Hepworth MR, Hardman MJ, Grencis RK (2010). The role of sex hormones in the development of Th2 immunity in a gender-biased model of Trichuris muris infection. Eur J Immunol.

[13] Bancroft AJ, Else KJ, Grencis RK (1994). Low-level infection with Trichuris muris significantly affects the polarization of the CD4 response. Eur J Immunol.

[14] Bancroft AJ, Else KJ, Humphreys NE, Grencis RK (2001). The effect of challenge and trickle Trichuris muris infections on the polarisation of the immune response. Int J Parasitol.

[15] Koyama K, Ito Y (1996). Comparative studies on immune responses to infection in susceptible B10.BR mice infected with different strains of the murine nematode parasite Trichuris muris. Parasite Immunol.

[16] Bellaby T, Robinson K, Wakelin D (1996). Induction of differential T-helper-cell responses in mice infected with variants of the parasitic nematode Trichuris muris. Infect Immun.

[17] D’Elia R, Behnke JM, Bradley JE, Else KJ (2009). Regulatory T cells: a role in the control of helminth-driven intestinal pathology and worm survival. J Immunol.

[18] Lee TD, Wakelin D, Grencis RK (1983). Cellular mechanisms of immunity to the nematode Trichuris muris. Int J Parasitol.

[19] Ito Y (1991). The absence of resistance in congenitally athymic nude mice toward infection with the intestinal nematode, Trichuris muris: resistance restored by lymphoid cell transfer. Int J Parasitol.

[20] Koyama K, Tamauchi H, Ito Y (1995). The role of CD4+ and CD8+ T cells in protective immunity to the murine nematode parasite Trichuris muris. Parasite Immunol.

[21] Koyama K (2002). NK1.1+ cell depletion in vivo fails to prevent protection against infection with the murine nematode parasite Trichuris muris. Parasite Immunol.

[22] Humphreys NE, Worthington JJ, Little MC, Rice EJ, Grencis RK (2004). The role of CD8+ cells in the establishment and maintenance of a Trichuris muris infection. Parasite Immunol.

[23] Else KJ, Grencis RK (1996). Antibody-independent effector mechanisms in resistance to the intestinal nematode parasite Trichuris muris. Infect Immun.

[24] Betts J, deSchoolmeester ML, Else KJ (2000). Trichuris muris: CD4+ T cell-mediated protection in reconstituted SCID mice. Parasitology.

[25] Svensson M, Russell K, Mack M, Else KJ (2010). CD4+ T-cell localization to the large intestinal mucosa during Trichuris muris infection is mediated by G alpha i-coupled receptors but is CCR6- and CXCR3-independent. Immunology.

[26] Little MC, Bell LV, Cliffe LJ, Else KJ (2005). The characterization of intraepithelial lymphocytes, lamina propria leukocytes, and isolated lymphoid follicles in the large intestine of mice infected with the intestinal nematode parasite Trichuris muris. J Immunol.

[27] Humphreys NE, Grencis RK (2002). Effects of ageing on the immunoregulation of parasitic infection. Infect Immun.

[28] Neill DR, McKenzie ANJ (2011). Nuocytes and beyond: new insights into helminth expulsion. Trends Parasitol.

[29] Saenz SA, Siracusa MC, Perrigoue JG, Spencer SP, Urban JF (2010). IL25 elicits a multipotent progenitor cell population that promotes TH2 cytokine responses. Nature.

[30] Neill DR, Wong SH, Bellosi A, Flynn RJ, Daly M (2010). Nuocytes represent a new innate effector leukocyte that mediates type-2 immunity. Nature.

[31] Moro K, Yamada T, Tanabe M, Takeuchi T, Ikawa T (2010). Innate production of TH2 cytokines by adipose tissue-associated c-Kit + Sca-1+ lymphoid cells. Nature.

[32] Price AE, Liang H-E, Sullivan BM, Reinhardt RL, Eisley CJ (2010). Systemically dispersed innate IL-13-expressing cells in type 2 immunity. Proc Natl Acad Sci.

[33] Perrigoue JG, Saenz SA, Siracusa MC, Allenspach EJ, Taylor BC (2009). MHC class II-dependent basophil-CD4+ T cell interactions promote TH2 cytokine-dependent immunity. Nat Immunol.

[34] Sokol CL, Barton GM, Farr AG, Medzhitov R (2008). A mechanism for the initiation of allergen-induced T helper type 2 responses. Nat Immunol.

[35] Sokol CL, Chu N-Q, Yu S, Nish SA, Laufer TM (2009). Basophils function as antigen-presenting cells for an allergen-induced T helper type 2 response. Nat Immunol.

[36] Wynn TA (2009). Basophils trump dendritic cells as APCs for TH2 responses. Nat Immunol.

[37] Siracusa MC, Saenz SA, Hill DA, Kim BS, Headley MB (2011). TSLP promotes interleukin-3-independent basophil haematopoiesis and type 2 inflammation. Nature.

[38] Kim S, Prout M, Ramshaw H, Lopez AF, LeGros G (2010). cutting edge: basophils are transiently recruited into the draining lymph nodes during helminth infection via IL-3, but infection-induced Th2 immunity can develop without Basophil lymph node recruitment or IL-3. J Immunol.

[39] Ohnmacht C, Schwartz C, Panzer M, Schiedewitz I, Naumann R (2010). Basophils orchestrate chronic allergic dermatitis and protective immunity against helminths. Immunity.

[40] Phythian-Adams AT, Cook PC, Lundie RJ, Jones LH, Smith KA (2010). CD11c depletion severely disrupts Th2 induction and development in vivo. J Exp Med.

[41] Sullivan BM, Liang H-E, Bando JK, Wu D, Cheng LE (2011). Genetic analysis of basophil function in vivo. Nat Immunol.

[42] Cruickshank SM, Deschoolmeester ML, Svensson M, Howell G, Bazakou A (2009). Rapid dendritic cell mobilization to the large intestinal epithelium is associated with resistance to Trichuris muris infection. J Immunol.

[43] MacDonald AS, Maizels RM (2008). Alarming dendritic cells for Th2 induction. J Exp Med.

[44] Coombes JL, Siddiqui KR, Arancibia-Carcamo CV, Hall J, Sun CM (2007). A functionally specialized population of mucosal CD103+ DCs induces Foxp3+ regulatory T cells via a TGF-beta and retinoic acid-dependent mechanism. J Exp Med.

[45] Sun CM, Hall JA, Blank RB, Bouladoux N, Oukka M (2007). Small intestine lamina propria dendritic cells promote de novo generation of Foxp3 T reg cells via retinoic acid. J Exp Med.

[46] Worthington JJ, Czajkowska BI, Melton AC, Travis MA (2011). Intestinal dendritic cells specialize to activate transforming growth factor-β and induce Foxp3+ regulatory T cells via integrin αvβ8. Gastroenterology.

[47] Mullaly SC, Burrows K, Antignano F, Zaph C (2011). Assessing the role of CD103 in immunity to an intestinal helminth parasite. PLoS One.

[48] Blackwell NM, Else KJ (2001). B cells and antibodies are required for resistance to the parasitic gastrointestinal nematode Trichuris muris. Infect Immun.

[49] Koyama K, Tamauchi H, Tomita M, Kitajima T, Ito Y (1999). B-cell activation in the mesenteric lymph nodes of resistant BALB/c mice infected with the murine nematode parasite Trichuris muris. Parasitol Res.

[50] Blackwell N, Else K (2002). A comparison of local and peripheral parasite-specific antibody production in different strains of mice infected with Trichuris muris. Parasite Immunol.

[51] Lee TD, Wakelin D (1982). The use of host strain variation to assess the significance of mucosal mast cells in the spontaneous cure response of mice to the nematode Trichuris muris. Int Arch Allergy Appl Immunol.

[52] Faulkner H, Renauld JC, Van Snick J, Grencis RK (1998). Interleukin-9 enhances resistance to the intestinal nematode Trichuris muris. Infect Immun.

[53] Koyama K, Ito Y (2000). Mucosal mast cell responses are not required for protection against infection with the murine nematode parasite Trichuris muris. Parasite Immunol.

[54] Betts CJ, Else KJ (1999). Mast cells, eosinophils and antibody-mediated cellular cytotoxicity are not critical in resistance to Trichuris muris. Parasite Immunol.

[55] Dixon H, Blanchard C, Deschoolmeester ML, Yuill NC, Christie JW (2006). The role of Th2 cytokines, chemokines and parasite products in eosinophil recruitment to the gastrointestinal mucosa during helminth infection. Eur J Immunol.

[56] Svensson M, Bell L, Little MC, DeSchoolmeester M, Locksley RM (2011). Accumulation of eosinophils in intestine-draining mesenteric lymph nodes occurs after Trichuris muris infection. Parasite Immunol.

[57] Else KJ, Finkelman FD, Maliszewski CR, Grencis RK (1994). Cytokine-mediated regulation of chronic intestinal helminth infection. J Exp Med.

[58] Bancroft AJ, Else KJ, Sypek JP, Grencis RK (1997). Interleukin-12 promotes a chronic intestinal nematode infection. Eur J Immunol.

[59] Helmby H, Takeda K, Akira S, Grencis RK (2001). Interleukin (IL)-18 promotes the development of chronic gastrointestinal helminth infection by downregulating IL-13. J Exp Med.

[60] Bancroft AJ, Humphreys NE, Worthington JJ, Yoshida H, Grencis RK (2004). WSX-1: a key role in induction of chronic intestinal nematode infection. J Immunol.

[61] Grencis RK, Entwistle GM (1997). Production of an interferon-gamma homologue by an intestinal nematode: functionally significant or interesting artefact?. Parasitology.

[62] Dénes Á, Humphreys N, Lane TE, Grencis R, Rothwell N (2010). Chronic systemic infection exacerbates ischemic brain damage via a CCL5 (regulated on activation, normal T-cell expressed and secreted)-mediated proinflammatory response in mice. J Neurosci.

[63] Grencis RK (2001). Cytokine regulation of resistance and susceptibility to intestinal nematode infection - from host to parasite. Vet Parasitol.

[64] Bancroft AJ, McKenzie AN, Grencis RK (1998). A critical role for IL-13 in resistance to intestinal nematode infection. J Immunol.

[65] Artis D, Humphreys NE, Bancroft AJ, Rothwell NJ, Potten CS (1999). Tumor necrosis factor alpha is a critical component of interleukin 13-mediated protective T helper cell type 2 responses during helminth infection. J Exp Med.

[66] Hayes KS, Bancroft AJ, Grencis RK (2007). The role of TNF-alpha in Trichuris muris infection II: global enhancement of ongoing Th1 or Th2 responses. Parasite Immunol.

[67] Richard M, Grencis RK, Humphreys NE, Renauld JC, Van Snick J (2000). Anti-IL-9 vaccination prevents worm expulsion and blood eosinophilia in Trichuris muris-infected mice. Proc Natl Acad Sci USA.

[68] Humphreys NE, Xu D, Hepworth MR, Liew FY, Grencis RK (2008). IL-33, a potent inducer of adaptive immunity to intestinal nematodes. J Immunol.

[69] Saenz SA, Noti M, Artis D (2010). Innate immune cell populations function as initiators and effectors in Th2 cytokine responses. Trends Immunol.

[70] Massacand JC, Stettler RC, Meier R, Humphreys NE, Grencis RK (2009). Helminth products bypass the need for TSLP in Th2 immune responses by directly modulating dendritic cell function. Proc Natl Acad Sci.

[71] Taylor BC, Zaph C, Troy AE, Du Y, Guild KJ (2009). TSLP regulates intestinal immunity and inflammation in mouse models of helminth infection and colitis. J Exp Med.

[72] Levison SE, McLaughlin JT, Zeef LAH, Fisher P, Grencis RK (2010). Colonic transcriptional profiling in resistance and susceptibility to trichuriasis: phenotyping a chronic colitis and lessons for iatrogenic helminthosis. Inflammatory Bowel Diseases.

[73] Summers RW, Elliott DE, Qadir K, Urban JF, Thompson R (2003). Trichuris suis seems to be safe and possibly effective in the treatment of inflammatory bowel disease. Am J Gastroenterol.

[74] Summers RW, Elliott DE, Urban JF, Thompson R, Weinstock JV (2005). Trichuris suis therapy in Crohn’s disease. Gut.

[75] Kradin RL, Badizadegan K, Auluck P, Korzenik J, Lauwers GY (2006). Iatrogenic Trichuris suis infection in a patient with Crohn disease. Arch Pathol Lab Med.

[76] DuPont AW, DuPont HL (2011). The intestinal microbiota and chronic disorders of the gut. Nat Rev Gastroenterol Hepatol.

[77] Schopf LR, Hoffmann KF, Cheever AW, Urban JF, Wynn TA (2002). IL-10 is critical for host resistance and survival during gastrointestinal helminth infection. J Immunol.

[78] Veldhoen M, Uyttenhove C, van Snick J, Helmby H, Westendorf A (2008). Transforming growth factor-beta “reprograms” the differentiation of T helper 2 cells and promotes an interleukin 9-producing subset. Nat Immunol.

[79] D’Elia R, Else KJ (2009). In vitro antigen presenting cell-derived IL-10 and IL-6 correlate with Trichuris muris isolate-specific survival. Parasite Immunol.

[80] La Flamme AC, MacDonald AS, Pearce EJ (2000). Role of IL-6 in directing the initial immune response to schistosome eggs. J Immunol.

[81] Collison LW, Chaturvedi V, Henderson AL, Giacomin PR, Guy C (2010). IL-35-mediated induction of a potent regulatory T cell population. Nat Immunol.

[82] Cliffe LJ, Potten CS, Booth CE, Grencis RK (2007). An increase in epithelial cell apoptosis is associated with chronic intestinal nematode infection. Infect Immun.

[83] Artis D, Potten CS, Else KJ, Finkelman FD, Grencis RK (1999). Trichuris muris: host intestinal epithelial cell hyperproliferation during chronic infection is regulated by interferon-gamma. Exp Parasitol.

[84] Cliffe LJ, Humphreys NE, Lane TE, Potten CS, Booth C (2005). Accelerated intestinal epithelial cell turnover: a new mechanism of parasite expulsion. Science.

[85] Bell LV, Else KJ (2011). Regulation of colonic epithelial cell turnover by IDO contributes to the innate susceptibility of SCID mice to Trichuris muris infection. Parasite Immunol.

[86] Artis D, Wang ML, Keilbaugh SA, He W, Brenes M (2004). RELMβ/FIZZ2 is a goblet cell-specific immune-effector molecule in the gastrointestinal tract. Proc Natl Acad Sci USA.

[87] Hasnain SZ, Wang H, Ghia J-E, Haq N, Deng Y (2010). Mucin gene deficiency in mice impairs host resistance to an enteric parasitic infection. Gastroenterology.

[88] Hasnain SZ, Evans CM, Roy M, Gallagher AL, Kindrachuk KN (2011). Muc5ac: a critical component mediating the rejection of enteric nematodes. J Exp Med.

[89] Hasnain SZ, Thornton DJ, Grencis RK (2011). Changes in the mucosal barrier during acute and chronic Trichuris muris infection. Parasite Immunol.

[90] Artis D, Wang ML, Keilbaugh SA, He W, Brenes M (2004). RELMbeta/FIZZ2 is a goblet cell-specific immune-effector molecule in the gastrointestinal tract. Proc Natl Acad Sci USA.

[91] Nair MG, Guild KJ, Du Y, Zaph C, Yancopoulos GD (2008). Goblet cell-derived resistin-like molecule beta augments CD4+ T cell production of IFN-gamma and infection-induced intestinal inflammation. J Immunol.

[92] Khan WI, Vallance BA, Blennerhassett PA, Deng Y, Verdu EF (2001). Critical role for signal transducer and activator of transcription factor 6 in mediating intestinal muscle hypercontractility and worm expulsion in Trichinella spiralis-infected mice. Infect Immun.

[93] Khan WI, Motomura Y, Blennerhassett PA, Kanbayashi H, Varghese AK (2005). Disruption of CD40-CD40 ligand pathway inhibits the development of intestinal muscle hypercontractility and protective immunity in nematode infection. Am J Physiol Gastrointest Liver Physiol.

[94] Khan WI, Richard M, Akiho H, Blennerhasset PA, Humphreys NE (2003). Modulation of intestinal muscle contraction by interleukin-9 (IL-9) or IL-9 neutralization: correlation with worm expulsion in murine nematode infections. Infect Immun.

[95] Motomura Y, Khan WI, El-Sharkawy RT, Verma-Gandhu M, Grencis RK (2010). Mechanisms underlying gut dysfunction in a murine model of chronic parasitic infection. Am J Physiol Gastrointest Liver Physiol.

[96] Albonico M, Engels D, Savioli L (2004). Monitoring drug efficacy and early detection of drug resistance in human soil-transmitted nematodes: a pressing public health agenda for helminth control. Int J Parasitol.

[97] Bergquist R, Lustigman S (2010) Chapter 10—control of important helminthic infections: vaccine development as part of the solution. Advances in parasitology. Academic Press. pp. 297–326. Available:http://www.sciencedirect.com/science/article/pii/S0065308X10730104. 10.1016/S0065-308X(10)73010-4 20627146

